# Whole-proteome phylogeny of large dsDNA viruses and parvoviruses through a composition vector method related to dynamical language model

**DOI:** 10.1186/1471-2148-10-192

**Published:** 2010-06-22

**Authors:** Zu-Guo Yu, Ka Hou Chu, Chi Pang Li, Vo Anh, Li-Qian Zhou, Roger Wei Wang

**Affiliations:** 1 School of Mathematical Sciences, Queensland University of Technology, GPO Box 2434, Brisbane, Q 4001, Australia; 2 School of Mathematics and Computational Science, Xiangtan University, Hunan 411105, China; 3 Department of Biology, The Chinese University of Hong Kong, Shatin, N.T., Hong Kong, China; 4 Department of Mathematics, The Chinese University of Hong Kong, Shatin, N.T., Hong Kong, China

## Abstract

**Background:**

The vast sequence divergence among different virus groups has presented a great challenge to alignment-based analysis of virus phylogeny. Due to the problems caused by the uncertainty in alignment, existing tools for phylogenetic analysis based on multiple alignment could not be directly applied to the whole-genome comparison and phylogenomic studies of viruses. There has been a growing interest in alignment-free methods for phylogenetic analysis using complete genome data. Among the alignment-free methods, a dynamical language (DL) method proposed by our group has successfully been applied to the phylogenetic analysis of bacteria and chloroplast genomes.

**Results:**

In this paper, the DL method is used to analyze the whole-proteome phylogeny of 124 large dsDNA viruses and 30 parvoviruses, two data sets with large difference in genome size. The trees from our analyses are in good agreement to the latest classification of large dsDNA viruses and parvoviruses by the International Committee on Taxonomy of Viruses (ICTV).

**Conclusions:**

The present method provides a new way for recovering the phylogeny of large dsDNA viruses and parvoviruses, and also some insights on the affiliation of a number of unclassified viruses. In comparison, some alignment-free methods such as the CV Tree method can be used for recovering the phylogeny of large dsDNA viruses, but they are not suitable for resolving the phylogeny of parvoviruses with a much smaller genome size.

## Background

Viruses were traditionally characterized by morphological features (capsid size, shape, structure, etc) and physicochemical and antigenic properties [1]. At the DNA level, the evolutionary relationships of many families and genera have been explored by sequence analysis of single gene or gene families, such as polymerase, capsid and movement genes [1]. The International Committee on the Taxonomy of Viruses (ICTV) publishes a report on the virus taxonomy system every five years. Phylogenetic and taxonomic studies of viruses based on complete genome data have become increasingly important as more and more whole viral genomes are sequenced [2-6]

The phylogeny based on single genes or gene families contains ambiguity because horizontal gene transfer (HGT), along with gene duplication and gene capture from hosts, appear to be frequent in large DNA viruses [7-10]. Whether single-gene based analysis can properly infer viral species phylogeny is debatable [2]. One of the unusual aspects of viral genomes is that they exhibit high sequence divergence [7,11]. Several works have attempted to infer viral phylogeny from their whole genomes [1,2,4,8,12-19]. Among these studies of genome trees, the alignment-free methods proposed by Gao and Qi [1], Wu et al [2], Gao et al [12] and Stuart et al [16] seem to be sufficiently powerful to resolve the phylogeny of viruses at large evolutionary distance. The present study represents another effort of applying an alignment-free method in analysing complete genome data to elucidate the phylogeny of two virus groups of different genome size, the large dsDNA viruses and parvoviruses.

The DNA of DNA viruses is usually double-stranded (dsDNA), but may also be single-stranded (ssDNA). According to the VIIIth Report of the International Committee on Taxonomy of Viruses (ICTV) [20], the dsDNA viruses can be classified into certain families or unassigned genus. The genome sizes of dsDNA viruses are usually larger than 10 kb except those in the families Polyomaviridae (5 kb) and Papillomaviridae (7-8 kb). On the other hand, the genome sizes of ssDNA viruses are smaller than 10 kb. The parvoviruses constitute a family established in 1970 to encompass all small non-enveloped viruses with approximately 5 kb linear, self-priming, ssDNA genomes [21,22]. According to the VIIIth Report of the International Committee on Taxonomy of Viruses (ICTV) [20], this family is separated into two subfamilies, Parvovirinae and Densovirinae. Viruses in the subfamily Parvovirinae infect vertebrates and vertebrate cell cultures, and frequently associate with other viruses, while those in the subfamily Densovirinae infect arthropods or other invertebrates [23,24]. Dependovirus requires co-infection with herpes or adenovirus for replication and is not itself pathogenic [22]. Due to the fatal nature of infection with densoviruses on their respective species, it has been suggested that densoviruses may represent suitable vectors for insect control [24,25]. The regions of identity and similarity between genomes of human and rodent parvoviruses and their respective hosts have been studied [26]. More features of parvoviruses can be found in the reviews by Tattersall and Cotmore [22].

The whole genome sequences are generally accepted as excellent tools for studying evolution [27]. On the basis of characters used to cluster genomes, Snel et al [28] reviewed that genome trees can be globally divided into five classes: alignment-free genome trees based on statistic properties of the complete genome, gene content trees based on the presence and absence of genes, genome trees based on chromosomal gene order, genome trees based on average sequence similarity, and phylogenomic trees based either on the collection of phylogenetic trees derived from shared gene families or on a concatenated alignment of those families. Due to the problems caused by the uncertainty in alignment [29], existing tools for phylogenetic analysis based on multiple alignment could not be directly applied to the whole-genome comparison and phylogenomic studies. There has been a growing interest in alignment-free methods for phylogenetic analysis using complete genome data [2,30,31]. Recently Jun et al [32] used an alignment-free method, the feature frequency profiles of whole proteomes, to construct a whole-proteome phylogeny of 884 prokaryotes and 16 unicellular eukaryotes. In their whole-proteome trees, Archaea, Eubacteria and Eukarya are clearly separated. Similarly, the analyses based on dynamical language (DL) model [33] and Markov model [34] without sequence alignment using 103 prokaryotes and six eukaryotes have yielded trees separating the three domains of life with the relationships among the taxa consistent with those based on traditional analyses. These two methods were also used to analyze the complete chloroplast genomes [33,35]. The CV Tree method [34] was recently used to analyze the fungal phylogeny [36]. A simplified version based on the CV Tree method was used to analyze gene sequencesfor the purpose of DNA barcoding [37,38]. Zheng et al [39] proposed a complexity-based measure for phylogenetic analysis. Guyon et al [40] compared four alignment-free string distances for complete genome phylogeny using 62 α-proteobacteria. The four distances are Maximum Significant Matches (MSM) distance, *K*-word (KW) or *K*-mer distance (i.e. the CV Tree method [33]), Average Common Substring (ACS) distance and Compression (ZL) distance. The results showed that the MSM distance outperforms the other three distances and the CV Tree method cannot give good phylogenetic topology for the 62 α-proteobacteria. We recently modified our dynamical language (DL) method [33] by replacing the correlation distance (pseudo-distance) by the chord distance (a proper distance in the strict mathematical sense) and proposed a way to select the optimal feature length based on average relative difference analysis [41]. Testing the modified DL method on the data sets used in previous studies [33,34,40], we found that this method can give very good phylogenetic topologies [41], while the CV tree method cannot give good phylogenetic topology for the 62 α-proteobacteria [40]. In the present paper, we adopt the DL method [33] to analyze a large number of genomes of the large dsDNA viruses and parvoviruses.

## Genome Data Sets

In order to explore the feasibility of our method, the whole DNA sequences (including protein-coding and non-coding regions), all protein-coding DNA sequences and all protein sequences from the complete genomes of the following two data sets were obtained from the NCBI genome database http://www.ncbi.nlm.nih.gov/genbank/genomes.

### **Data set 1 **(used in [1])

We selected 124 large dsDNA viruses. The species in the family Adenoviridae are: *Bovine adenovirus *D (BAdV_4, NC_002685), *Ovine adenovirus *D (OAdV_D, NC_004037), *Duck adenovirus *A (DAdV_A, NC_001813), *Fowl adenovirus *A (FAdV_A, NC_001720) and *Fowl adenovirus *D (FAdV_D, NC_000899) in the genus *Atadenovirus*; *Bovine adenovirus *B (BAdV_B, NC_001876), *Canine adenovirus *(CAdV, NC_001734), *Human adenovirus *A (HAdV_A, NC_001460), *Human adenovirus *B (HAdV_B, NC_004001), *Human adenovirus *C (HAdV_C, NC_001405), *Human adenovirus *D (HAdV_D, NC_002067), *Human adenovirus *E (HAdV_E, NC_003266), *Murine adenovirus *A (MAdV_A, NC_000942), *Ovine adenovirus *A (OAdV_A, NC_002513), *Porcine adenovirus *C (PAdV_C, NC_002702), *Simian adenovirus *A (SAdV_3, NC_006144), *Bovine adenovirus *A (BAdV_A, NC_006324), *Human adenovirus *F (HAdV_F, NC_001454), *Porcine adenovirus *A (PAdV_A, NC_005869), *Tree shrew adenovirus *(TSAdV, NC_004453) and *Simian adenovirus *1 (SAdV_1, NC_006879) in the genus *Mastadenovirus*; *Frog adenovirus *(FrAdV, NC_002501) and *Turkey adenovirus *A (TAdV_A, NC_001958) in the genus *Siadenovirus*. In the family Asfarviridae, we only selected the *African swine fever virus *(ASFV, NC_001659) in the genus *Asfivirus*. The viruses in the family Baculoviridae are: *Adoxophyes orana granulovirus *(AdorGV, NC_005038), *Agrotis segetum granulovirus *(AsGV, NC_005839), *Cryptophlebia leucotreta granulovirus *(CrleGV, NC_005068), *Cydia pomonella granulovirus *(CpGV, NC_002816), *Phthorimaea operculella granulovirus *(PhopGV, NC_004062), *Plutella xylostella granulovirus *(PlxyGV, NC_002593) and *Xestia c-nigrum granulovirus *(XecnGV, NC_002331) in genus *Granulovirus*; *Autographa californica nucleopolyhedrovirus *(AcMNPV, NC_001623), *Bombyx mori nucleopolyhedrovirus *(BmNPV, NC_001962), *Choristoneura fumiferana defective nucleopolyhedrovirus *(CfDeFNPV, NC_005137), *Choristoneura fumiferana MNPV *(CfMNPV, NC_004778), *Epiphyas postvittana nucleopolyhedrovirus *(EppoNPV, NC_003083), *Helicoverpa armigera nuclear polyhedrosis virus *(HearNPV, NC_003094), *Helicoverpa armigera nucleopolyhedrovirus G4 *(HearNPVG4, NC_002654), *Helicoverpa zea single nucleocapsid nucleopolyhedrovirus *(HzSNPV, NC_003349), *Lymantria dispar nucleopolyhedrovirus *(LdMNPV, NC_001973), *Mamestra configurata nucleopolyhedrovirus A *(MacoNPV_A, NC_003529), *Mamestra configurata nucleopolyhedrovirus B *(MacoNPV_B, NC_004117), *Neodiprion sertifer nucleopolyhedrovirus *(NeseNPV, NC_005905), *Orgyia pseudotsugata multicapsid nucleopolyhedrovirus *(OpMNPV, NC_001875), *Rachiplusia ou multiple nucleopolyhedrovirus *(RoMNPV, NC_004323), *Spodoptera exigua nucleopolyhedrovirus *(SeMNPV, NC_002169) and *Spodoptera litura nucleopolyhedrovirus *(SpltNPV, NC_003102) in genus *Nucleopolyhedrovirus*; and two unclassified viruses *Culex nigripalpus baculovirus *(CuniNPV, NC_003084), *Neodiprion lecontei nucleopolyhedrovirus *(NeleNPV, NC_005906). The species in the family Herpesviridae are: *Gallid herpesvirus 1 *(GaHV_1, NC_006623) in genus *Iltovirus*; *Gallid herpesvirus 2 *(GaHV_2, NC_002229), *Gallid herpesvirus 3 *(GaHV_3, NC_002577) and *Meleagrid herpesvirus 1 *(MeHV_1, NC_002641) in genus *Mardivirus*; *Meleagrid herpesvirus 1 *(MeHV_1, NC_002641), *Cercopithecine herpesvirus 1 *(CeHV_1, NC_004812), *Human herpesvirus 1 *(HHV_1, NC_001806), *Human herpesvirus 2 *(HHV_2, NC_001798) and *Cercopithecine herpesvirus 2 *(CeHV_2, NC_006560) in genus *Simplexvirus*; *Bovine herpesvirus 1 *(BoHV_1, NC_001847), *Bovine herpesvirus 5 *(BoHV_5, NC_005261), *Cercopithecine herpesvirus 9 *(CHV_7, NC_002686), *Equid herpesvirus 1 *(EHV_1, NC_001491), *Equid herpesvirus 4 *(EHV_4, NC_001844), *Suid herpesvirus 1 *(SuHV_1, NC_006151) and *Human herpesvirus *3 (strain Dumas) (HHV_3, NC_001348) in genus *Varicellovirus*; *Human herpesvirus *5 strain AD169 (HHV5L, NC_001347), *Human herpesvirus *5 strain Merlin (HHV5w, NC_006273), *Pongine herpesvirus 4 *(PoHV_4, NC_003521) and *Cercopithecine herpesvirus 8 *(CeHV_8, NC_006150) in genus *Cytomegalovirus*; *Murid herpesvirus 1 *(MuHV_1, NC_004065) and *Murid herpesvirus 2 *(MuHV_2, NC_002512) in genus *Muromegalovirus*; *Human herpesvirus 6 *(HHV_6, NC_001664), *Human herpesvirus 6B *(HHV_6B, NC_000898) and *Human herpesvirus 7 *(HHV_7, NC_001716) in genus *Roseolovirus*; *Callitrichine herpesvirus 3 *(CalHV_3, NC_004367), *Human herpesvirus 4 *(HHV_4, NC_009334) and *Cercopithecine herpesvirus 15 *(CeHV_15, NC_006146) in genus *Lymphocryptovirus*; *Cercopithecine herpesvirus 17 *(CeHV_17, NC_003401), *Alcelaphine herpesvirus 1 *(AIHV_1, NC_002531), *Bovine herpesvirus 4 *(BoHV_4, NC_002665), *Equid herpesvirus 2 *(EHV_2, NC_001650), *Human herpesvirus 8 *(HHV_8, NC_003409), *Murid herpesvirus 4 *(MuHV_4, NC_001826) and *Saimiriine herpesvirus 2 *(SaHV_2, NC_001350) in genus *Rhadinovirus*; *Ictalurid herpesvirus 1 *(IcHV_1, NC_001493) in genus *Ictalurivirus*; and 4 unassigned species *Tupaiid herpesvirus 1 *(TuHV_1, NC_002794), *Ostreid herpesvirus 1 *(OsHV_1, NC_005881), *Psittacid herpesvirus 1 *(PsHV_1, NC_005264) and *Ateline herpesvirus 3 *(AtHV_3, NC_001987). The species in the family Iridoviridae are: *Invertebrate iridescent virus 6 *(IIV_6, NC_003038) in genus *Iridovirus*; *Lymphocystis disease virus *- isolate China (LCDV_IC, NC_005902) and *Lymphocystis disease virus 1 *(LCDV_1, NC_001824) in genus *Lymphocystivirus*; *Infectious spleen and kidney necrosis virus *(ISaKNV, NC_003494) in genus *Megalocytivirus*; *Frog virus 3 *(FV_3, NC_005946), *Regina ranavirus *(ATV, NC_005832) and *Singapore grouper iridovirus *(SiGV, NC_006549) in genus *Ranavirus*. In the family Nimaviridae, we only selected *Shrimp white spot syndrome virus *(WSSV, NC_003225) in genus *Whispovirus*. The two species in the family Phycodnaviridae are *Paramecium bursaria Chlorella virus 1 *(PBCV_1, NC_000852) in genus *Chlorovirus *and *Ectocarpus siliculosus virus *(EsV_1, NC_002687) in genus *Phaeovirus*. The two species in the family Polydnaviridae are *Cotesia congregata virus *(CcBV, NC_006633-62) and *Microplitis demolitor bracovirus *(MdBV, NC_007028-41) in genus *Bracovirus*. The species in family Poxviridae are: *Canarypox virus *(CNPV, NC_005309) and *Fowlpox virus *(FWPV, NC_002188) in genus *Avipoxvirus*; *Lumpy skin disease virus *(LSDV, NC_003027) and *Sheeppox virus *(SPPV, NC_004002) in genus *Capripoxvirus*; *Myxoma virus *(MYXV, NC_001132) and *Rabbit fibroma virus *(SFV, NC_001266) in genus *Leporipoxvirus*; *Molluscum contagiosum virus *(MOCV, NC_001731) in genus *Molluscipoxvirus*; *Camelpox virus *(CMLV, NC_003391), *Cowpox virus *(CPXV, NC_003663), *Ectromelia virus *(ECTV, NC_004105), *Monkeypox virus *(MPXV, NC_003310), *Vaccinia virus *(VACV, NC_006998) and *Variola virus *(VARV, NC_001611) in genus *Orthopoxvirus*; *Bovine papular stomatitis virus *(BPSV, NC_005337) and *Orf virus *(ORFV, NC_005336) in genus *Parapoxvirus*; *Swinepox virus *(SWPV, NC_003389) in genus *Suipoxvirus*; *Yaba monkey tumor virus *(YMTV, NC_005179) and *Yaba-like disease virus *(YDV, NC_002642) in genus *Yatapoxvirus*; *Amsacta moorei entomopoxvirus *(AMEV, NC_002520) and *Melanoplus sanguinipes entomopoxvirus *(MSEV, NC_001993) in genus *Betaentomopoxvirus*; and unclassified *Mule deer poxvirus *(DPV, NC_006966). There are another two viruses *Acanthamoeba polyphaga mimivirus *(APMiV, NC_006450) in genus *Mimivirus *(unassigned to a family) and *Heliothis zea virus 1 *(HZV_1, NC_004156) (unclassified).

### **Data set 2 **(selected from Table one in [24] and Table three in [42])

We selected 30 parvoviruses. There are 20 species in the subfamily Parvovirinae and 10 species in the subfamily Densovirinae. The species in the subfamily Parvovirinae are: *Aleutian mink disease virus *(ADMV, NC_001662) in the genus *Amdovirus*; *Minute virus of canines *(MVC, NC_004442) in the genus *Bocavirus*; *Adeno-associated virus *1 (AAV1, NC_002077), *Adeno-associated virus *2 (AAV2, NC_001401), *Adeno-associated virus *3 (AAV3, NC_001729), *Adeno-associated virus *4 (AAV4, NC_001829), *Adeno-associated virus *5 (AAV5, NC_006152), *Adeno-associated virus *7 (AAV7, NC_006260), *Adeno-associated virus *8 (AAV8, NC_006261), *Avian adeno-associated virus *ATCC VR-865 (AAAVa, NC_004828), *Avian adeno-associated virus *strain DA-1 (AAAVd, NC_006263), *Bovine adeno-associated virus *(BAAV, NC_005889), *Bovine parvovirus*-2 (BPV2, NC_006259), *Goose parvovirus *(GPV, NC_001701) and *Muscovy duck parvovirus *(MDPV, NC_006147) in the genus *Dependovirus*; *B19 virus *(B19V, NC_000883) in the genus *Erythrovirus*; *Canine parvovirus *(CPV, NC_001539), *LuIII parvovirus *(LuIIIV, NC_004713), *Mouse parvovirus *3 (MPV, NC_008185) and *Porcine parvovirus *(PPV, NC_001718) in the genus *Parvovirus*. The species in the subfamily Densovirinae are: *Aedes albopictus densovirus *(AalDNV, NC_004285) in the genus *Brevidensovirus*; *Acheta domesticus densovirus *(AdDNV, NC_004290), *Diatraea saccharalis densovirus *(DsDNV, NC_001899), *Galleria mellonella densovirus *(GmDNV, NC_004286), *Junonia coenia densovirus *(JcDNV, NC_004284) and *Mythimna loreyi densovirus *(MIDNV, NC_005341) in the genus *Densovirus*; *Bombyx mori densovirus *1 (BmDNV1, NC_003346), *Bombyx mori densovirus *5 (BmDNV5, NC_004287) and *Casphalia extranea densovirus *(CeDNV, NC_004288) in the genus *Iteravirus*; and *Periplaneta fuliginosa densovirus *(PfDNV, NC_000936) in the genus *Pefudensovirus*. The genera ***Amdovirus ***and ***Bocavirus***, and the genus ***Pefudensovirus ***are newly defined genera in the subfamilies **Parvoririnae **and Densovirinae respectively in the VIIIth Report of ICTV [12]. We also notice that AAV7, AAV8, AAAVa, BPV2, MPV, AdDNV and CeDNV are still unclassified in the VIIIth Report of ICTV.

### Remark

The words in the brackets given above are the abbreviations of the names of these species and their NCBI accession numbers.

## Results and Discussion

**Figure 1:**
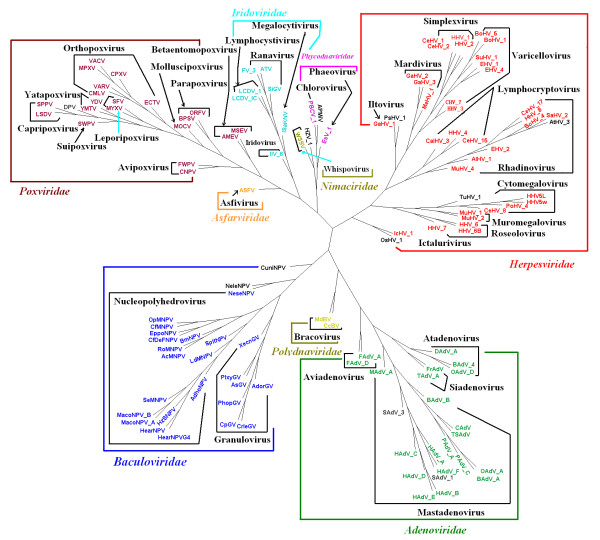
**The NJ tree of 124 large dsDNA virus genomes based on the all protein sequences using the DL method for *K *= 5**.

**Figure 2:**
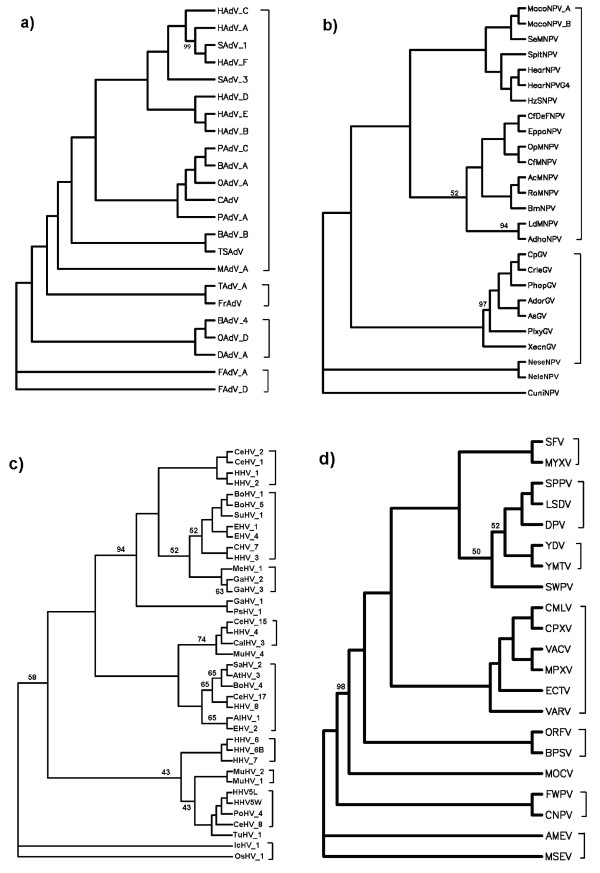
**The bootstrap consensus trees for the four big groups in Figure 1 based on 100 replicates, a): Adenoviridae, b): Baculoviridae, c): Herpesviridae, d): Poxviridae**. Modified bootstrap percentages <100% are shown (other branches are 100% supported).

**Figure 3:**
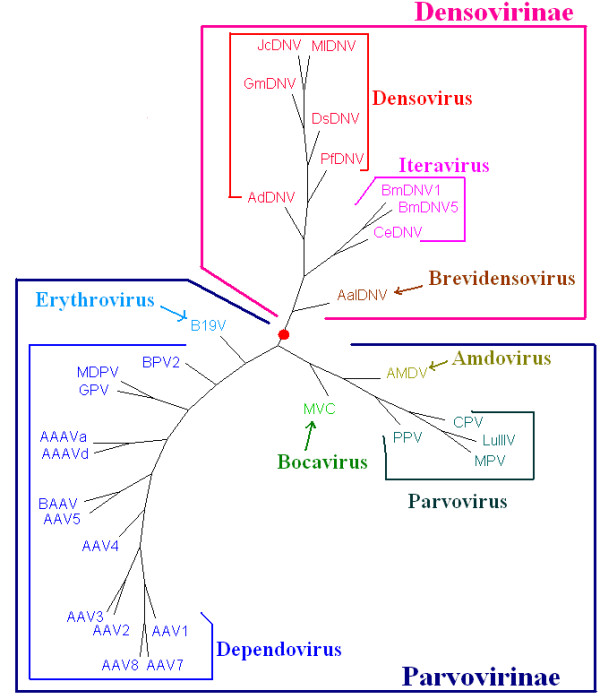
**The NJ tree of 30 parvovirus genomes based on the all protein sequences using the modified DL method for *K *= 4**.

**Figure 4:**
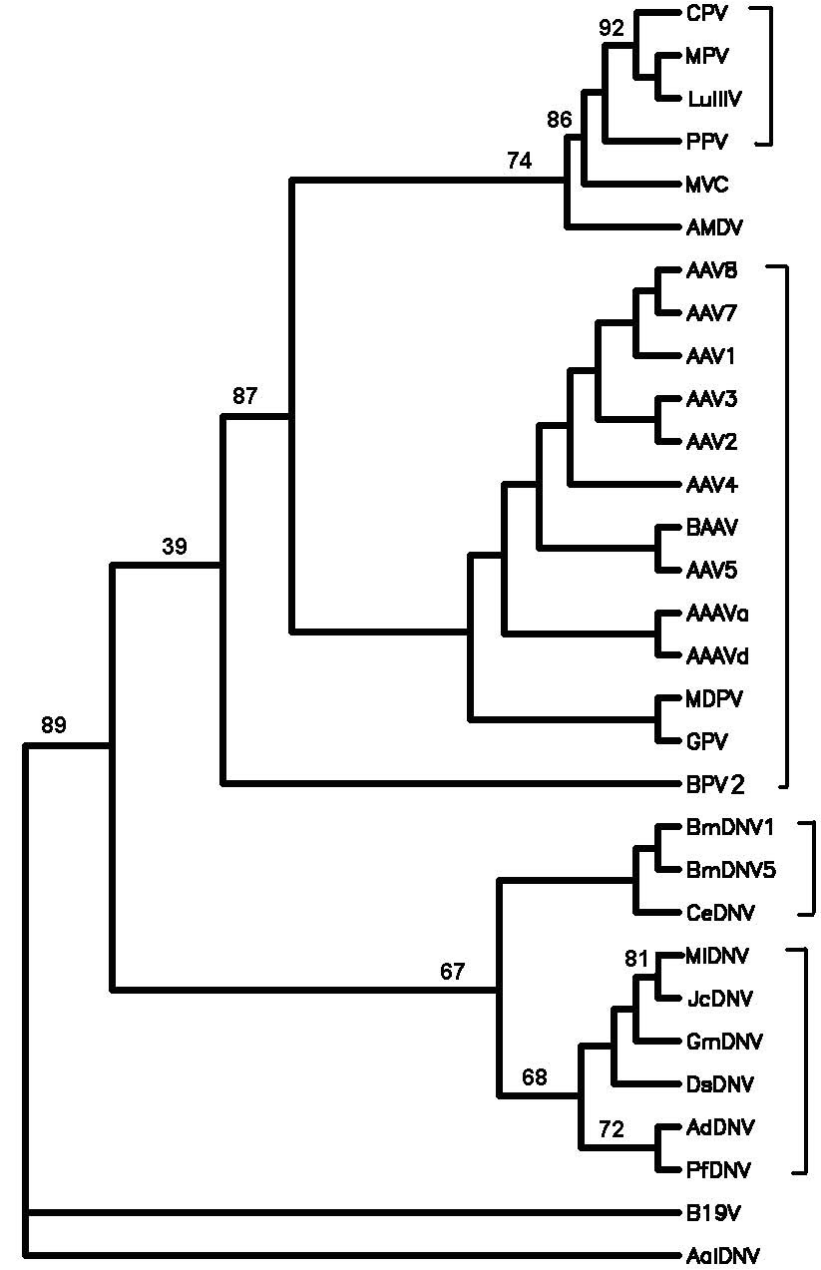
**The bootstrap consensus trees for Figure 3 based on 100 replicates**. Modified bootstrap percentages <100% are shown (other branches are 100% supported).

The whole DNA sequences, all protein-coding DNA sequences and all protein sequences from complete genomes of the selected 124 large dsDNA viruses and 30 selected parvoviruses were analyzed. The trees of *K *= 3 to 6 based on all protein sequences and the trees of *K *≤ 13 based on the whole DNA sequences and all protein-coding DNA sequences using the DL method [33] were constructed. After comparing all the trees constructed by the present method with the classification of the 124 large dsDNA viruses and 30 parvoviruses given in the VIIIth Report of ICTV [23], we found that the trees for large dsDNA viruses and parvoviruses based on all protein sequences are better than those based on all protein-coding DNA sequences and the whole DNA sequences. Furthermore, for the phylogenetic trees of 124 large dsDNA viruses based on all protein sequences, the tree of *K *= 5 provides the best result among the cases of *K *= 3 to 6. We show this tree in Figure 1. The trees for *K *= 4 and 6 are similar to but a little bit inferior to the tree for *K *= 5. The bootstrap consensus trees for the four big groups (Adenoviridae, Baculoviridae, Herpesviridae and Poxviridae) (Figure 2) provide branch statistics for the tree in Figure 1. For the trees of 30 parvoviruses based on all protein sequences, the trees for *K *= 4 and 6 are topologically identical, and are the best trees among the cases of *K *= 3 to 6. We show the tree for *K *= 4 in Figure 3. The tree for *K *= 5 is similar to but a little bit worse than the trees for *K *= 4 and 6. Figure 4 shows the bootstrap consensus tree of Figure 3. The distance matrices generated from our analyses are available from the first author via email yuzg1970@yahoo.com. We found that the DL method [33] and the modified DL method [41] give trees of the same topology for the same *K *for both data sets.

As given in Figure 1, despite numerous horizontal gene transfers among large dsDNA viruses [9], our analysis can divide the 124 dsDNA viruses into nine families correctly. Our phylogenetic relationships of all 124 large dsDNA viruses are in good agreement with the latest classification in the VIIIth Report of the International Committee on Taxonomy of Viruses (ICTV) [20]. In the family Adenoviridae, Figures 1 and 2a support the division of this family into four genera *Atadenovirus*, *Aviadenovirus*, *Mastadenovirus *and *Siadenovirus*. All viruses in these four genera are grouped correctly. The topology of phylogeny for these four genera is identical to that shown in Figure one of [1] which supports the hypothesis that interspecies transmission, i.e. host switches of adenoviruses, may have occurred [42]. In Figures 1 and 2b, the family Baculoviridae is divided into two genera *Granulovirus *and *Nucleopolyhedrovirus*. All viruses in these two genera are classified correctly. The unclassified virus NeleNPV in this family groups with NeseNPV which belongs to genus *Nucleopolyhedrovirus*. So our result supports grouping virus NeleNPV to genus *Nucleopolyhedrovirus*. Another unclassified virus CuniNPV is located at the basal position of this family, as reported by Herniou et al [43], with the Hymenoptera baculoviruses (NeleNPV and NeseNPV) and Lepidoptera baculoviruses (the remaining species) grouped together, as reported by Herniou et al [8] and Zanotto et al [44]. Thus the classification of CuniNPV is still unresolved in our analysis. The division of the family Poxviridae into two subfamilies Chordopoxvirinae and Entomopoxvirinae shown in Figures 1 and 2d is the same as in the VIIIth Report of ICTV. In the subfamily Chordopoxvirinae, the viruses in the genera *Avipoxvirus, Capripoxvirus, Leporipoxvirus, Molluscipoxvirus, Orthopoxvirus, Parapoxvirus, Suipoxvirus *and *Yatapoxvirus *group together correctly. The unclassified virus DPV is closely related to the genera *Capripoxvirus *and *Suipoxvirus*, so that our result supports assigning the virus DPV to the subfamily Chordopoxvirinae, in agreement with the results in [1] and [45]. In the subfamily Entomopoxvirinae, the viruses in genus Betaentomopoxvirus group together as expected. The division of the family Herpesviridae into subfamilies Alphaherpesvirinae, Gammaherpesvirinae and Betaherpesvirinae is clear. All viruses in the genera within each subfamily are grouped correctly in Figures 1 and 2c. Consistent with the result of [1], our tree supports assigning the unclassified virus TuHV_1 in the subfamily Betaherpesvirinae to genus *Cytomegalovirus*. The unclassified Herpesviridae virus OsHV_1 groups with IcHV_1 indicating that we can assign it to the genus *Ictalurivirus*. The unclassified Herpesviridae virus PsHV_1 groups with GaHV_1, suggesting its affiliation to the genus *Iltovirus*. The unclassified Herpesviridae virus AtHV_3 nests inside the branch of genus *Rhadinovirus*, which indicates we can assign it to the genus *Rhadinovirus*. All viruses in the family Iridoviridae fall into their genera correctly in Figure 1. The grouping of the unclassified virus HZV_1 with WSSV indicates its affiliation to the genus *Whispovirus *in the family Nimaviridae. The viruses in family Phycodnaviridae group together as expected. The virus APMiV of the genus *Mimivirus *but with no family affiliation nests within the family Phycodnaviridae suggests classification of the genus to this family. The viruses in the family Polydnaviridae cluster together correctly. As claimed by Gao and Qi [1], our results could also provide some clues to the hypotheses on the origins and evolution of viruses of several families. Overall, the topology of our tree is similar to that of the tree in [1] and our tree is slightly better *because the tree in *[1]*has 4 outliers *(CuniNPV, IIV_6, IcHV_1 and OsHV_1) *while ours has no outlier*. Although the results using feature frequency profiles (FFPs) on a slightly larger data set showed the FFP method can also resolve the phylogeny of large dsDNA viruses [2], the optimal feature length for FFP method is 8 implying that much longer computing time and larger computer space are needed as compared to our method with an optimal feature length of 5.

As shown in Figures 3 and 4, our analyses showed that the parvovirus genomes are separated into two major groups, with one group corresponding to the subfamily Parvovirinae and the other group corresponding to the subfamily Densovirinae. In the Parvovirinae group, the parvoviruses in the genera *Parvovirus*, *Erythrovirus*, *Dependovirus*, *Amdovirus *and *Bocavirus *group together as subgroups respectively. In the Densovirinae group, the parvoviruses in the genera *Densovirus*, *Iteravirus *and *Brevidensovirus *cluster together as subgroups correctly. All the groups and subgroups shown in Figure 3 using our method agree well with the latest classification of parvoviruses given in the VIIIth Report of ICTV [20] except PfDNV. PfDNV was classified into the *Brevidensovirus *in the VIIth Report of the ICTV [23] and reclassified into *Pefudensovirus *in the VIIIth Report of ICTV [20]. After the comparison on the genome structure, coding protein sequence homology, DNA sequence homology, 3-dimensional structure [46,47] between PfDNV and other parvoviruses, Li et al [48] claimed that it would be more appropriate to classify pfDNV as *Densovirus *rather than *Brevidensovirus*. Thus the grouping of PfDNV with *Densoviru *in our tree (Figures 3 and 4) provides another piece of evidence for classifying PfDNV as *Densovirus*. *Amdovirus *and *Bocavirus *are newly defined genera in the subfamily Parvoririnae in the VIIIth Report of ICTV [20]. In the VIIth Report of ICTV [23], the parvoviruses (AMDV and MVC) in these two new genera were grouped under *Parvovirus*. Their close relationship is also reflected in our trees (Figures 3 and 4) in which *Amdovirus *and *Bocavirus *cluster with *Parvovirus *as a separate branch. The parvoviruses AAV7, AAV8, AAAVa, BPV2, MPV, AdDNV and CeDNV are still not classified in the VIIIth Report of ICTV [20]. In our previous study [33], the DL method applied to the analysis of 103 prokaryotes and six eukaryotes has yielded trees separating the three domains of life, Archaea, Eubacteria and Eukarya with the relationships among the taxa in good agreement with those based on traditional analyses. It has also been applied in analyzing the chloroplast genomes [33] to give reliable phylogenies of plants and algae. From the above discussion, it is clear that this method can successfully resolve the phylogeny of parvoviruses. The positions of AAV7, AAV8, AAAVa, BPV2, MPV, AdDNV and CeDNV in Figure 3 provide new insights on their classification.

It is very interesting to note the assumption that small DNA viruses (genome size <10 kb) probably have different evolutionary history as compared to large DNA viruses [9,49]. Our analyses showed that the DL method can be used to reconstruct the phylogeny of viruses with large difference in genome size (larger than 10 kb for large dsDNA viruses and less than 10 kb for parvoviruses). We also generated all the trees of the same *K *values based on the three kinds of sequences for the parvovirus data set 2 using the CV Tree method [34]. Yet no tree generated by the CV Tree method can clearly distinguish the subfamilies Parvovirinae and Densovirinae of parvoviruses. So for the data set of parvoviruses, the DL method is superior (from the biological point of view) to the CV Tree method in phylogenetic inference.

Our approach is faster than the traditional approaches of phylogenetic analysis, particularly when dealing with a large number of genomes. Moreover, since multiple sequence alignment is not necessary, the intrinsic problems associated with this complex procedure can be avoided. Our method may provide a quick reference on virus phylogeny and a fast analysis of co-evolution of viruses and their hosts when their proteomes are available [1,50].

## Conclusions

Using the DL method, we have studied the molecular phylogeny between families of large dsDNA viruses and parvoviruses. The present method provides a new way for recovering the phylogeny of large dsDNA viruses and parvoviruses, and also insights on the affiliation of some unclassified viruses and relationships among some families. It appears that some alignment-free methods such as the CV Tree method [34] can be used for recovering the phylogeny of large dsDNA viruses, but they are not suitable for parvoviruses with a much smaller genome size.

## Methods

In this paper, three kinds of data from the complete genomes of large dsDNA viruses and parvoviruses are analysed using the DL method proposed by our group [33]. They are the whole DNA sequences (including protein-coding and non-coding regions), all protein-coding DNA sequences and the amino acid sequences of all protein-coding genes.

There are a total of *N *= 4^*K*^(for DNA sequences) or 20^*K*^(for protein sequences) possible types of *K*-mers (the strings with fixed length *K*). We denote the length of a DNA or protein sequence as *L*. Then a window of length *K *is used to slide through the sequences by shifting one position at a time to determine the frequencies of each of the *N *kinds of *K*-mers in this sequence. We define *p*(*α*_1_*α*_2_...*α*_*K*_) = *n*(*α*_1_*α*_2_...*α*_*K*_)/(*L *- *K *+ 1) as the observed frequency of a *K*-mer *α*_1_*α*_2_...*α*_*K*_, where *n*(*α*_1_*α*_2_...*α*_*K*_) is the number of times that *α*_1_*α*_2_...*α*_*K *_appears in this sequence. For the DNA or amino acid sequences of the protein-coding genes, denoting by *m *the number of protein-coding genes from each complete genome, we define  as the observed frequency of a *K*-mer *α*_1_*α*_2_...*α*_*K*_; here *n*_*j*_(*α*_1_*α*_2_...*α*_*K*_) means the number of times that *α*_1_*α*_2_...*α*_*K *_appears in the *j*th protein-coding DNA sequence or protein sequence, and *L*_*j *_the length of the *j*th sequence in this complete genome. Then we can form a composition vector for a genome using *p*(*α*_1_*α*_2_...*α*_*K*_) as components for all possible *K*-mers *α*_1_*α*_2_...*α*_*K*_. We use *p*_*i *_to denote the *i*-th component corresponding to the mer type *i*, *i *= 1,..., *N (N *mers are arranged in a fixed order as the alphabetical order). In this way we construct a composition vector *p *= (*p*_1_, *p*_2_,..., *p*_N_) for a genome.

Yu et al [33] considered an idea from the theory of dynamical language that a *K*-mer *s*_1_*s*_2_...*s*_*K *_is possibly constructed by adding a letter *s*_*K *_to the end of the (*K*-1) -mer *s*_1_*s*_2_...*s*_*K*-1_or a letter *s*_1 _to the beginning of the (*K*-1)-mer *s*_2_*s*_3_...*s*_*K*_. After counting the observed frequencies for all (*K*-1)-mers and the four or 20 kinds of letters, the expected frequency of appearance of *K*-mers is predicted by

where *p*(*s*_1_) and *p*(*s*_*K*_)are frequencies of nucleotides or amino acids *s*_1_and *s*_*K *_appearing in this genome. Then *q*(*s*_1_*s*_2_...*s_K_) of all 4^*K *^or 20^*K*^K*-mers is viewed as the noise background. We then subtract the noise background by defining

The transformation *X *= (*p*/*q*)-1 has the desired effect of subtraction of random background in *p *and rendering it a stationary time series suitable for subsequent cross-correlation analysis. *X *can also be regarded as the relative difference between *p *and *q*.

Then we use *X*(*s*_1_*s*_2_...*s*_*K*_) for all possible *K*-mers *s*_1_*s*_2_...*s*_*K *_as components and arrange them according to a fixed alphabetical order to form a composition vector *X *= (*X*_1_,*X*_2_,...,*X*_*N*_) for genome *X*, and likewise *Y *= (*Y*_1_,*Y*_2_,...,*Y*_*N*_) for genome *Y*.

Then we view the *N *components in the vectors *X *and *Y *as samples of these two random variables respectively. The sample correlation *C*(*X*, *Y*) between any two genomes *X *and *Y*is defined in the usual way. The dissimilarity *D*(*X, Y*) between the two genomes is then defined by *D*(*X, Y*) = (1 - *C*(*X*, *Y*))/2. A dissimilarity matrix for all the genomes under study is then generated for the construction of phylogenetic trees. This method to construct phylogenetic tree is referred to as the dynamical language (DL) method [33].

Finally, for convenience to compare the results with those of the previous works, based on the distance matrices, we construct all trees for data sets 1 and 2 using the neighbour-joining (NJ) method [51] in the softwares Phylip [52] (version 3.66) and *SplitsTree4 *[51] (version 4.10).

### Robustness test of the trees using modified version of the bootstrap method [2]

In order to estimate the robustness of tree topology, Qi et al [34] proposed a bootstrap method by resampling on the genes or translated proteins on the genome (this method was also used by Wang et al [36]). Wu et al [2] proposed a modified bootstrap method by resampling the frequencies of all *K*-mers. The method of Qi et al. [34] is not applicable to the virus genomes as almost all virus genomes have only a few genes and it is statistically meaningless to resample such a small number of genes. So we use the modified bootstrap method proposed by Wu et al [2] which works as follows. A table is first constructed with each row being the composition vector representing a genome and each column representing the frequencies of a fixed *K*-mer in different genomes. The bootstrap is applied to the columns of the table except those are redrawn [2]. Thus, the resampled table has fewer columns but each *K*-mer maintains the same frequency as in the original table. Because it is allowed that some positions could be redrawn more than once in the traditional bootstrap analyses with sequence alignment [53], we believe it is more reasonable to allow that some columns to be redrawn more than once, giving the resampled table with the same number of columns as the original table in the modified bootstrap method. Then a distance matrix can be obtained based on the resampled table.

## Authors' contributions

ZGY conceived the study, downloaded the data, analyzed the results, and was involved in programming, drafting and revising the manuscript. KHC and CPL participated in designing the study, analyzed the results, and were involved in drafting and revising the manuscript. VVA, LQZ and WRW participated in the discussion of the results, and were involved in revising the manuscript. All authors read and approved the final manuscript.
